# Generation of structure-based pharmacophores using energetic analysis – application on fragment docking

**DOI:** 10.1186/1758-2946-3-S1-O20

**Published:** 2011-04-19

**Authors:** Kathryn Loving, Noeris Salam, Daniel Cappel, Woody Sherman

**Affiliations:** 1 Schrödinger Inc. 120 West 45th Street, 17th Floor, New York, NY 10036-4041, USA; 2 Schrödinger GmbH, Dynamostr. 13, Mannheim, 68165, Germany

## 

We describe a novel method to develop energetically optimized, structure-based pharmacophores for use in rapid in silico screening. The method combines pharmacophore perception and database screening with protein ligand energetic terms computed by the Glide XP scoring function to rank the importance of pharmacophore features. We derive energy-optimized pharmacophore hypotheses for 30 pharmaceutically relevant crystal structures and screen a database to assess the enrichment of active compounds. The method is compared to three other approaches: (1) pharmacophore hypotheses derived from a systematic assessment of receptor ligand contacts, (2) Glide SP docking, and (3) 2D ligand fingerprint similarity. The method developed here shows better enrichments than the other three methods and yields a greater diversity of actives than the contact-based pharmacophores or the 2D ligand similarity. We then apply this method on fragment docking using fragment-specific settings aimed to generate poses that explore every pocket of a binding site while maintaining the docking accuracy of the top scoring pose. Next, we describe how the energy terms from the Glide XP scoring function are mapped onto pharmacophore sites from the docked fragments in order to rank their importance for binding. We show that the most energetically favourable pharmacophore sites are consistent with features from known tight binding compounds. The derived pharmacophore models are able to recover known active compounds from a database screen and retrieving diverse compounds that are not biased by the co-crystallized ligand.

